# Simultaneous Quantification of Four Marker Compounds in *Bauhinia coccinea* Extract and Their Potential Inhibitory Effects on Alzheimer’s Disease Biomarkers

**DOI:** 10.3390/plants10040702

**Published:** 2021-04-06

**Authors:** Yu Jin Kim, Eunjin Sohn, Hye-Sun Lim, Yoonju Kim, Joo-Hwan Kim, Soo-Jin Jeong

**Affiliations:** 1 Clinical Medicine Division, Korea Institute of Oriental Medicine, Daejeon 34054, Korea; jinjin0228@kiom.re.kr (Y.J.K.); ssen4022@kiom.re.kr (E.S.); qp1015@kiom.re.kr (H.-S.L.); pray4u96@kbri.re.kr (Y.K.); 2 Department of Life Science, Gachon University, Seongnam 13120, Korea; kimjh2009@gachon.ac.kr

**Keywords:** *Bauhinia coccinea*, simultaneous quantification, neuroprotection, antioxidation, acetylcholinesterase, amyloid-β

## Abstract

*Bauhinia coccinea* is a tropical woody plant widely distributed in Vietnam and Unnan in southern China. Although many studies have shown the biological activities of extracts from various other species in the genus, no studies have investigated the effects of *B. coccinea* extracts on biological systems. In the present study, a quantitative analysis of four marker compounds of ethanol extracts of *B. coccinea* branches (EEBC) was performed using the high performance liquid chromatography (HPLC)-photodiode array (PDA) method. Among gallic acid, (+)-catechin, ellagic acid, and quercitrin contained in EEBC, the most abundant compound was (+)-catechin (18.736 mg/g). In addition, we investigated the EEBC on neuroprotection, antioxidation, and Alzheimer’s disease (AD) marker molecules, acetylcholinesterase (AChE), and amyloid-β (Aβ). EEBC significantly inhibited hydrogen peroxide (H_2_O_2_)-induced cell death in a HT22 neuronal cell line and increased 2,2′-azino-bis(3-ethylbenzothiazoline-6-sulfonic acid) and 2,2-diphenyl-1-picrylhydrazyl scavenging activity markedly. EEBC also inhibited AChE and Aβ aggregation. Among the four compounds, gallic acid exhibited strong inhibitory effects against AChE activation. In the Aβ aggregation assay, the four marker compounds exhibited inhibitory effects lower than 30%. According to the results, EEBC could exert anti-AChE activation and Aβ aggregation activities based on the interactive effects of the marker compounds. Our findings suggest that EEBC are sources of therapeutic candidates for application in the development of AD medication based on AChE and Aβ dual targeting.

## 1. Introduction

Aging is an irreversible phenomenon and a potential severe risk factor for the incidence of various chronic diseases, including neurodegenerative, cardiovascular, and metabolic diseases, in addition to cancers [1]. Among age-related diseases, neurodegenerative diseases, especially Alzheimer’s disease (AD), are becoming major public health burdens as the elderly patient population increases rapidly. AD is a brain disease in which neuronal cell death causes memory and cognitive impairment. Neuronal cells are more susceptible to oxidative stress owing to their high oxygen consumption rates, which generates excess reactive oxygen species (ROS) [2]. Numerous studies have investigated anti-AD therapeutics based on the control of the redox system [3,4]. In addition, numerous investigations have considered the activities of key biomarkers, acetylcholinesterase (AChE) activation, and amyloid-β (Aβ) aggregation, as bio-targets in the development of anti-AD drugs [5,6,7]. Despite the extensive efforts to develop AD therapies, synthetic compounds have certain limitations. For example, adverse effects of Food and Drug Administration-approved AChE inhibitors (donepezil, galantamine, rivastigmine, and tacrine) have been reported, including nausea, vomiting, muscle cramps, bradycardia, and gastric acid overproduction [8,9]. Therefore, natural materials with potent antioxidant activities, such as medicinal plants or phytochemicals, have been proposed as alternative sources of therapies [10,11,12].

*Bauhinia coccinea* (Lour.) DC. belongs to the Fabaceae family and is also known as *Phanera coccinea* [13]. There are more than 500 species of flowering plants in the genus *Bauhinia* within the Cercidoideae subfamily [14]. Plants in the genus *Bauhinia* have been shown to have various pharmacological effects. For example, *B. purpurea* extracts possess anti-arthritic [15], antiulcer, antisecretory, cytoprotective [16], analgesic, and anti-inflammatory properties [17]. In addition, *B. forficata* extracts exhibit hypoglycemic and antioxidant activities in vivo [18,19,20], while *B. championii* flavone extracts exhibit antioxidant, anti-inflammatory, and anti-apoptotic activities [21]. The anti-inflammatory and anti-apoptotic effects of *B. championii* were assessed using myocardial ischemia/reperfusion (I/R) injury rat models. In anti-inflammatory assays, *B. championii* restricted the release of inflammatory mediators and inhibited the toll-like receptor 4 (TLR4)/nuclear factor kappa B (NF-κB) signaling pathway. *B. championii* also showed anti-apoptotic activity by reducing the elevated Bax/Bcl-2 ratios and caspase-3 activation. Previous phytochemical studies on *Bauhinia* spp. have revealed the presence of phenylpropanoids, flavanones, bibenzyls, and dihydrodibenzoxepins in *B. purpurea* [22,23], flavonoids and diterpenoids in *B. championii* [24,25], and flavonoids in *B. curvula* [26]. However, no pharmacological studies on the effects of *B. coccinea* have yet been reported.

In the present study, we report the potential application of ethanol extracts of *B. coccinea* branches (EEBC) as novel anti-AD therapies for the first time. We investigated the antioxidant activity and the inhibitory effects of the extracts on AChE and butyrylcholinesterase (BChE) activities, and Aβ aggregation. We also performed quantitative analyses of four marker compounds from *B. coccinea* branches using high performance liquid chromatography (HPLC) analyses and carried out in vitro AChE and Aβ aggregation assays using the marker compounds.

## 2. Results

### 2.1. Optimization of HPLC Condition

**Figure 1:**
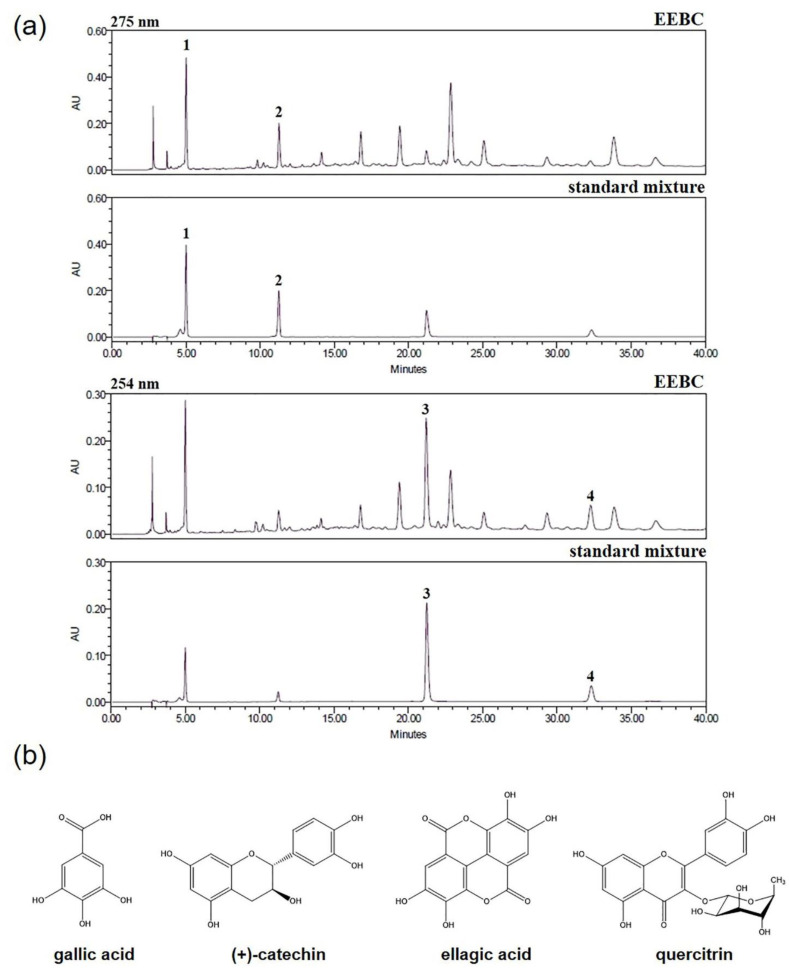
(**a**) HPLC chromatograms of ethanol extracts of *B. coccinea* branches (EEBC) and standard mixture at 275 and 254 nm. Gallic acid (1), (+)-catechin (2), ellagic acid (3), and quercitrin (4); (**b**) Chemical structures of the four marker compounds in *B. coccinea*. HPLC: high performance liquid chromatography. EEBC: ethanol extracts of *B. coccinea* branches.

The HPLC analytical method was established for the simultaneous separation of the four marker compounds in the EEBC. The four compounds were successfully separated within 33 min using two mobile phases consisting of 0.1% (*v*/*v*) aqueous trifluoroacetic acid (TFA) and acetonitrile and detected at 275 nm (gallic acid and (+)-catechin) and 254 nm (ellagic acid and quercitrin). The retention times of gallic acid, (+)-catechin, ellagic acid, and quercitrin were 4.99, 11.25, 21.18, and 32.23 min, respectively. The HPLC chromatograms for the EEBC and the standard mixture are presented in Figure 1a. The chemical structures of the compounds are illustrated in Figure 1b.

### 2.2. Regression Equation, Linearity, Limits of Detection (LOD), and Limits of Quantification (LOQ)

The linear relationships between the peak area (*y*) and concentration (*x*, µg/mL) of each compound are represented by the regression equations (*y* = a*x* + b) in Table 1. The calibration curves for the four compounds showed good linearity (*r*^2^ = 1.0000). The LOD and LOQ of the four marker compounds were 0.172–0.726 µg/mL and 0.52–2.199 µg/mL, respectively.

### 2.3. Quantitative Analysis of the Four Marker Compounds in EEBC

The established HPLC-photodiode array (PDA) analytical method was applied for the simultaneous quantification of the four marker compounds in EEBC. The amounts of the four marker compounds ranged from 2.912 to 18.736 mg/g, and the results are listed in Table 2. Among the four compounds, (+)-catechin (18.736 mg/g) was the most abundant.

### 2.4. Protective Effects of EEBC in Hydrogen Peroxide (H_2_O_2_)-Induced Neuronal Cell Damage

**Figure 2:**
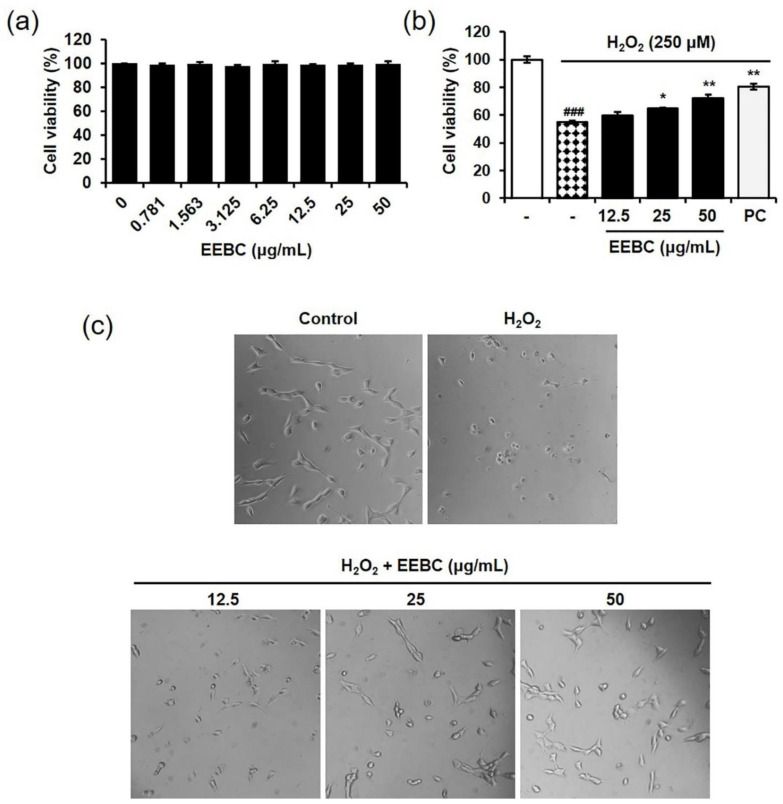
Protective effects of EEBC against H_2_O_2_-damaged HT22 neuronal cells. (**a**) Cells were incubated with various concentrations of EEBC for 24 h and cell viability evaluated using a Cell Counting Kit (CCK) assay; (**b**) Cells were co-treated with EEBC (0, 12.5, 25, or 50 μg/mL) and H_2_O_2_ (250 μM) for 6 h. CCK assay was performed to assess changes in cell viability. Caveolin was used as a positive control. Data are expressed as % of the control. Values represent mean ± SEM. ^###^ *p* < 0.001 vs. untreated cells; * *p* < 0.05 and ** *p* < 0.01 vs. H_2_O_2_-treated cells; (**c**) Morphological changes in cells were observed under inverted microscopy (Eclipse TS100, Nikon, Japan). EEBC: ethanol extract of *B. coccinea* branches. CCK: Cell Counting Kit. PC: positive control.

Many studies have reported the neuroprotective effects of (+)-catechin, gallic acid, ellagic acid, and quercitrin, the marker compounds of EEBC [27,28,29,30]. However, no study has investigated if *B. coccinea* has protective effects against neuronal damage. We first investigated the cytotoxicity of EEBC in HT22 murine hippocampal cells. Cells were treated with EEBC at concentrations ranging from 0 to 50 μg/mL for 24 h. According to the results, EEBC had no significant toxicity in HT22 cells (Figure 2a). To induce neuronal cell death, HT22 cells were exposed to H_2_O_2_. As illustrated in Figure 2b, H_2_O_2_ treatment reduced cell viability significantly when compared to the untreated control. To explore the protective effects of EEBC, various concentrations of EEBC (0, 12.5, 25, or 50 μg/mL) were co-treated with H_2_O_2_. EEBC blocked H_2_O_2_-induced cell death significantly at 25 and 50 μg/mL. Consistent with the results of the cell viability assay, H_2_O_2_ treatment induced morphological features associated with damaged cells, whereas co-treatment with H_2_O_2_ and EEBC prevented the adverse morphological changes (Figure 2c).

### 2.5. Antioxidant Activity of EEBC via Free Radical Scavenging Actions

**Figure 3:**
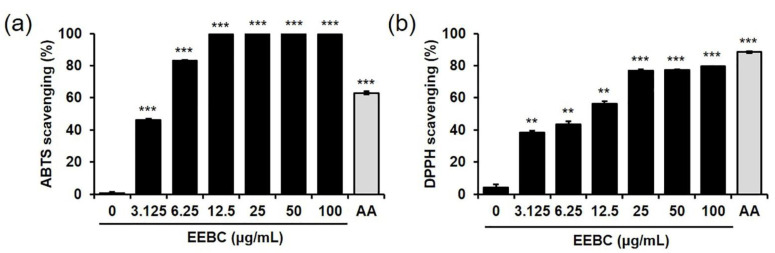
Free radical scavenging activity of EEBC. (**a**) Various concentrations of EEBC (0, 3.125, 6.25, 12.5, 25, 50, or 100 μg/mL) were reacted with equal volumes of ABTS solution for 5 min in the dark at room temperature. Absorbance of the reactants was read at 734 nm using a spectrophotometer; (**b**) Various concentrations of EEBC (0, 3.125, 6.25, 12.5, 25, 50, or 100 μg/mL) were reacted with equal volumes of DPPH solution for 30 min in the dark at room temperature. Absorbance of the reactants was read at 517 nm using a spectrophotometer. Ascorbic acid (AA, 5 μg/mL) was used as a positive control. Each value is presented as the mean ± SEM. ** *p* < 0.01 and *** *p* < 0.001 vs. control. EEBC: ethanol extract of *Bauhinia coccinea* branches. ABTS: 2,2′-azino-bis(3-ethylbenzothiazoline-6-sulfonic acid). DPPH: 2,2-diphenyl-1-picrylhydrazyl.

Oxidative stress in neuronal cells is a major target in the development of therapeutic drugs for various neurodegenerative diseases [31]. We explored the EEBC antioxidant activities by measuring the free radical scavenging activity against 2,2′-azino-bis(3-ethylbenzothiazoline-6-sulfonic acid (ABTS) and 2,2-diphenyl-1-picrylhydrazyl (DPPH). As shown in Figure 3a,b, EEBC increased both ABTS and DPPH scavenging activities dramatically, indicating the antioxidant activity of EEBC. EC_50_ values of EEBC in ABTS and DPPH scavenging assays were 3.37 and 6.63 μg/mL, respectively.

### 2.6. Effects of EEBC and Its Marker Compounds on AD Biomarkers

**Figure 4:**
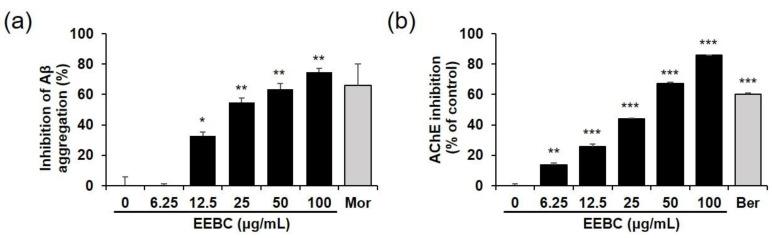
Effects on EEBC on amyloid-β (Aβ) aggregation and acetylcholinesterase (AChE) activity in vitro. (**a**) Various concentrations of EEBC (0, 6.25, 12.5, 25, 50, or 100 μg/mL) were mixed with Aβ (1–42), followed by the addition of 10 µL of thioflavin T dye. Fluorescence of thioflavin T was measured at intervals of 20 min for 2 h, with an excitation wavelength (λ_ex_) of 440 nm and an emission wavelength (λ_em_) of 485 nm on a SpectraMax i3 Multi-Mode Detection Platform. Morin (100 μM) was used as the positive control; (**b**) Various concentrations of EEBC (0, 6.25, 12.5, 25, 50, or 100 μg/mL) were mixed with the substrates acetylthiocholine iodide and DTNB, and incubated for 10 min at room temperature. AChE solution was then added to the initial mixture and incubated again for 1 h at room temperature. Absorbance was measured at 412 nm using an Epoch microplate spectrophotometer. Berberine (0.5 μM) was used as a positive control. Each value is presented as the mean ± SEM. * *p* < 0.05, ** *p* < 0.01, or *** *p* < 0.001 vs. control. EEBC: ethanol extract of *B. coccinea* branches. Aβ: amyloid-β. AChE: acetylcholinesterase. DTNB: 5,5′-dithiobis-2-nitrobenzoic acid.

In the present study, Aβ aggregation, and AChE and BChE activity assays were conducted to investigate the influence of EEBC on AD pathogenesis. EEBC markedly increased the inhibition of both Aβ aggregation and AChE activity in a dose-dependent manner (Figure 4a,b, respectively). IC_50_ values of EEBC in Aβ aggregation and AChE activity assays were 28.60 and 27.71 μg/mL, respectively. However, there was no BChE inhibitory effect even at the 100 μg/mL, whereas the inhibitory activity of the positive control berberine (50 μM) reached 57% (Table S1).

**Figure 5:**
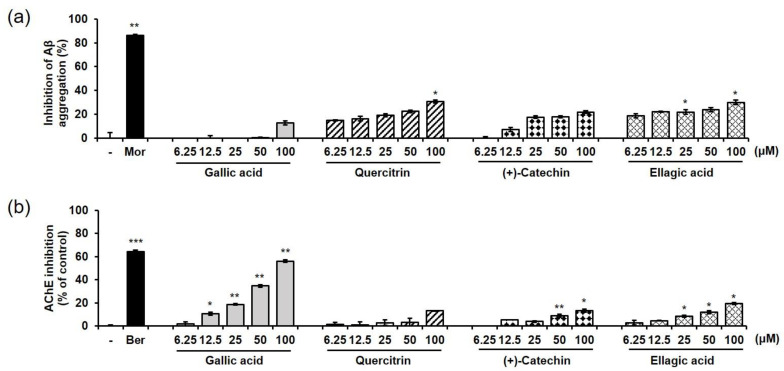
Effects of marker EEBC compounds on Aβ aggregation and AChE activity. (**a**) For the in vitro Aβ aggregation assay, gallic acid, quercitrin, (+)-catechin, and ellagic acid (6.25, 12.5, 25, 50, or 100 μM) were mixed with Aβ (1–42), followed by the addition of 10 µL of thioflavin T dye. Fluorescence of thioflavin T was measured at intervals of 20 min for 2 h, with an excitation wavelength (λ_ex_) of 440 nm and an emission wavelength (λ_em_) of 485 nm on a SpectraMax i3 Multi-Mode Detection Platform. Morin (100 μM) was used as the positive control; (**b**) For the in vitro AChE activity assay, gallic acid, quercitrin, (+)-catechin, and ellagic acid (6.25, 12.5, 25, 50, or 100 μM) were mixed with the substrates, acetylthiocholine iodide and DTNB, and incubated for 10 min at room temperature. AChE solution was then added to the initial mixture and additionally incubated for 1 h at room temperature. Absorbance was measured at 412 nm using an Epoch microplate spectrophotometer. Berberine (0.5 μM) was used as the positive control. Each value is presented as the mean ± SEM. * *p* < 0.05, ** *p* < 0.01, or *** *p* < 0.001 vs. control. EEBC: ethanol extract of *B. coccinea* branches. Aβ: amyloid-β. AChE: acetylcholinesterase. DTNB: 5,5′-dithiobis-2-nitrobenzoic acid.

In addition, we tested the effects of four marker compounds of EEBC on Aβ aggregation, and AChE and BChE activities. Gallic acid, quercitrin, (+)-catechin, and ellagic acid increased the ratio of inhibition of Aβ aggregation in a dose-dependent manner. Quercitrin and ellagic acid exhibited approximately 30% inhibition of the Aβ aggregation at 100 μM. Gallic acid and (+)-catechin inhibited Aβ aggregation by 12.6% and 22.0% at 100 μM, respectively, but had no significant effect (Figure 5a). In the AChE activity assay, gallic acid exhibited the highest inhibitory activity (56.1% at 100 μM, IC_50_ = 82.44 μM) among the four marker compounds. The AChE inhibition of (+)-catechin and ellagic acid was lower than 20% and that of quercitrin had no significant effect (Figure 5b). In the BChE activity assay, all four marker compounds had no inhibitory effect (Table S1).

## 3. Discussion

AD is the most common neurodegenerative disease, and the associated memory and cognitive impairment severely interfere with normal daily life in patients. One of the AD risk factors is increasing age; the majority of patients with AD are aged over 65 years [32]. Clinical signs of AD are thought to be associated with neuronal loss or cell death in the brain [33]. In addition to neuronal changes, several pathological phenomena are observed, such as Aβ accumulation or aggregation and AChE enzymatic activation [34]. Most current AD therapies target AChE and may temporarily improve symptoms or delay the progression of the disease; however, they have no curative effects [35]. To overcome the limitations of AChE inhibitors, numerous research groups have recently attempted to develop novel AD therapies to target other AD biomarkers such as Aβ [36]. However, sustained failures of clinical trials have been a disappointment for both patients with AD and medical staff [37]. Consequently, it is essential to consider “the complex and multifactorial nature of AD” in AD drug development. Recently, drug discovery approaches have shifted from single-target to multi-target paradigms [38].

Plants have diverse compounds in their tissues, some of which have medicinal properties. Therefore, multi-compound and multi-target activities of plant compounds have major benefits for drug development for complex diseases such as AD. In addition, oxidative damage is increasingly considered a precursor to Aβ accumulation in AD progression [39,40]. Therefore, the widely recognized antioxidant activities in most plants could facilitate the development of AD drugs. In the present study, we carried out a simultaneous analysis of four marker compounds, gallic acid, (+)-catechin, ellagic acid, and quercitrin, in EEBC, using the HPLC-PDA method. Gallic acid, (+)-catechin, ellagic acid, and quercitrin were well separated at 4.99, 11.25, 21.18, and 32.23 min retention times, respectively. The amounts of the four compounds ranged between 2.912 and 18.736 mg/g, and the most abundant compound was (+)-catechin (18.736 mg/g).

In our investigation, the capacity of EEBC to target multiple factors associated with AD pathogenesis was evaluated. First, we explored whether EEBC exhibits protective effects against neuronal cell death. Oxidative stress arising from ROS overproduction plays a critical role in neurodegeneration and neuronal loss [41]. We induced oxidative stress-mediated neuronal cell death by exposing HT22 hippocampal cells to H_2_O_2_. HT22 is an immortalized murine hippocampal neuronal cell line subcloned from the HT-4 cells that originally immortalized from primary mouse hippocampal tissues [42,43]. In many studies, HT22 cells are used as a valuable in vitro model to investigate the neuronal cell death [44,45,46]. Notably, EEBC treatment inhibited H_2_O_2_-induced neuronal cell death significantly, in a dose-dependent manner. Along with the neuroprotective effects, antioxidant EEBC activities were demonstrated by scavenging of ABTS and DPPH free radicals. Accumulating evidence supports the potential of various phytochemicals to attenuate oxidative neurotoxicity. Some of the plant sources of such phytochemicals include *Zizyphus lotus* [47], *Ginkgo biloba* [48], and *Eugenia dysenterica* [49], while some phytochemicals include (-)-epigallocatechin-3-gallate, resveratrol, curcumin, and quercetin [50].

Aβ is generated from amyloid precursor protein (APP) by cleavage with β- and γ-secretases successively. Cleaved Aβ peptides are subsequently aggregated and accumulated in the brain, leading to oxidative stress and neuronal cell death [51]. Reduced acetylcholine level in a damaged brain is another hallmark of AD development. Numerous studies are still exploring mechanisms of targeting cholinergic dysfunction as potential AD therapies, to achieve efficacies greater than those of current AChE inhibitor drugs [52]. Previous studies reported evaluation of the cholinesterase (ChE) inhibitory effects of genus *Bauhinia* and their chemical components. For instance, flowers of *B. variegata*, *B.* var. *candida*, and *B. ungulata* exhibited inhibitory activity against the AChE by retention factor analysis based on the thin layer chromatography technique [53]. 7,4′-Dihydroxyflavone isolated from the stems of *B. pentandra* inhibited AChE activity [54]. In addition, dihydroquercetin in the bark of *B. variegata* was reported as a good candidate for AD treatment by binding to the active sites of AChE and BChE in molecular docking and molecular dynamics simulations [55]. Notably, several studies have suggested that AChE accelerates the assembly of Aβ peptides into fibrils in the brain [56]. Additionally, AChE interacts with Aβ aggregates in the brain of patients with AD. In a related study, Carvajal and Inestrosa reported that IDN5706, a hyperforin derivative, inhibits the interaction between AChE and Aβ [57]. Duan et al. also reported that silibinin could act as a dual target inhibitor of AChE and Aβ in AD treatment [58].

In the present study, the effects of EEBC on Aβ aggregation, and AChE and BChE activation were evaluated. EEBC markedly increased the inhibition of both Aβ aggregation and AChE activity in a dose-dependent manner. We also examined the inhibitory effects of four EEBC marker compounds on Aβ aggregation, and AChE and BChE activities. Four marker compounds, gallic acid, quercitrin, (+)-catechin, and ellagic acid, exhibited moderate inhibitory activity against Aβ aggregation (≤30% inhibition) compared to high EEBC inhibitory activity (75% inhibition, IC_50_ = 28.60 μg/mL). In contrast to the results of the Aβ assay, gallic acid had higher inhibitory activity against AChE than the other compounds, quercitrin, (+)-catechin, and ellagic acid. However, gallic acid also exhibited lower inhibitory effects against AChE (IC_50_ = 82.44 μM) when compared to EEBC (IC_50_ = 27.71 μg/mL). In addition, since gallic acid had no inhibitory effect on BChE at 100 μM, it exhibited selectivity for inhibition of AChE over BChE. The results imply that EEBC efficacy in targeting AD biomarkers could be demonstrated based on synergistic interactions among various compounds constituting EEBC. Additionally, only four compounds were identified, and several peaks are still unidentified especially at 17, 19, and 23 min in HPLC analysis. Of undetected compounds, one or more might mainly contribute to inhibitory effects of EEBC on the Aβ aggregation and AChE activation.

Additional investigations are required to determine the bioactive compounds in EEBC that are responsible for targeting AChE and Aβ. We performed HPLC analysis to identify marker compounds of EEBC and observed more than 10 peaks on a HPLC chromatogram, including identified and unidentified compounds. In the future, we will determine the unidentified compounds by isolating each peak and analyzing the chemical structures of the compounds using a nuclear magnetic resonance system. Following the identification of all major marker compounds of EEBC, we would be able to determine the potential bioactive compounds in EEBC that inhibit AD biomarkers based on in vitro and in vivo experiments and investigate the molecular mechanisms responsible for anti-AD effects of EEBC. Overall, our results highlight the multi-targeting capacity of EEBC on AD-related neurodegenerative changes, including neuroprotection, inhibition of Aβ aggregation, and AChE inhibition.

## 4. Materials and Methods

### 4.1. Plant Materials

The *B. coccinea* branches used in the present study were provided by the Korean Seed Association and identified by Professor Joo-Hwan Kim (Gachon University, Seongnam, Korea). Voucher specimen (SCD-A-115) has been deposited at the Herbarium, Korea Institute of Oriental Medicine (Daejeon, Korea).

### 4.2. Chemicals and Reagents

The four marker compounds, gallic acid (CFN99624), (+)-catechin (CFN99646), ellagic acid (CFN98716), and quercitrin (CFN98850), were purchased from ChemFaces Biochemical Co., Ltd. (Wuhan, China). The purity of the marker compounds was ≥98.0%, as assessed using HPLC analysis. The solvents, acetonitrile and water, which were used for analyses, were purchased from J. T. Baker Chemical Co. (Phillipsburg, NJ, USA), and the reagent, TFA was purchased from Sigma-Aldrich (St. Louis, MO, USA).

### 4.3. Preparation of Sample and Standard Solutions

The dried *B. coccinea* branches (30 g) were cut into small pieces and extracted three times with 70% aqueous ethanol (300 mL) at room temperature for 7 days. The extracted solution was filtered through a filter paper (5 µm) and concentrated using a rotary evaporator (EYELA N-1000, Rikakikai Co., Tokyo, Japan) under vacuum to obtain a powdered extract (5.66 g). The yield of the EEBC was 18.86%. The EEBC was weighed accurately, dissolved in methanol at a concentration of 10 mg/mL, and filtered through a syringe filter (0.45 µm) for quantitative analysis.

The four marker compounds were weighed and dissolved in methanol at 1.0 mg/mL. The stock solutions were diluted with methanol to yield a series of standard solutions for use in quantitative analyses.

### 4.4. Apparatus and Chromatographic Conditions

To identify and quantify the four compounds in *B. coccinea*, a Waters Alliance e2695 system (Waters Corp., Milford, MA, USA) consisting of a pump, an auto sample injector, a column oven, and a PDA detector (2998; Waters Corp.) was used. The ultraviolet (UV) wavelength range of the PDA detector was 190–400 nm. The data were acquired and processed using Empower software (version 3, Waters Corp., Milford, MA, USA). Chromatographic separation of the four compounds was performed using a Sunfire C_18_ analytical reversed-phase column (250 × 4.6 mm, 5 µm, Waters Corp) maintained at 40 °C, with the mobile phase consisting of 0.1% (*v*/*v*) aqueous TFA (A) and acetonitrile (B) forming a gradient elution of 7–16% B for 0–10 min, 16–20% B for 10–45 min, 20–100% B for 45–50 min, and 100% B for 50–60 min. The flow rate of the mobile phase was 1 mL/min, and the sample injection volume was 10 µL.

### 4.5. Calibration Curve and Limits of Detection and Quantification

The calibration curves of the four marker compounds were calculated from the peak areas of the standard solutions at different concentrations. The concentration ranges of gallic acid, (+)-catechin, ellagic acid, and quercitrin were 3.125–100 µg/mL, 12.5–400 µg/mL, 3.125–100 µg/mL, and 6.25–200 µg/mL, respectively. The solutions were measured in triplicate for the calibration curves. The LOD and LOQ for the four marker compounds were calculated using the slopes of the calibration curves and the standard deviations (SD) of the responses, using the following equations:

LOD = 3.3 × (SD of the response/Slope of the calibration curve),(1)

LOQ = 10 × (SD of the response/Slope of the calibration curve).(2)

### 4.6. Cell Culture and Drug Treatment

HT22 cells were obtained from Merck Millipore (Darmstadt, Germany). HT22 cells were maintained in Dulbecco’s Modified Eagle’s medium (Hyclone/Thermo, Rockford, IL, USA) supplemented with 10% fetal bovine serum (Hyclone/Thermo, Rockford, IL, USA) and penicillin/streptomycin in 5% CO_2_ at 37 °C. HT22 cells were co-treated with EEBC and H_2_O_2_ (500 μM, Sigma-Aldrich, St. Louis, MO, USA) for 6 h.

### 4.7. Cell Viability Assay

The cytotoxic effects of EEBC against HT22 cells were evaluated using the cell counting kit-8 (CCK-8) assay. HT22 cells were plated on 96-well microplates at a density of 3 × 10^4^/well and treated with various concentrations of EEBC for 24 h. CCK-8 solution (Dojindo, Kumamoto, Japan) was added, and the cells were incubated for 4 h. The absorbance was measured at 450 nm on an Epoch Microplate Spectrophotometer (BioTek Instruments, Inc., Winooski, VT, USA).

Cell viability (%) = (Mean OD in EEBC–treated cells/Mean OD in untreated cells) × 100(3)

### 4.8. Free Radical Scavenging Assay

ABTS radical cations were produced by reacting 7 mM ABTS solution with 2.45 mM potassium persulfate in the dark at room temperature for 16 h. Absorbance of the reactant was later adjusted to 0.7, at a wavelength of 734 nm. Different concentrations (6.25–200 µg/mL) of 100 μL aliquots of EEBC solution were mixed with 100 μL ABTS^•+^ solution. The reaction mixtures were incubated for 5 min in the dark at room temperature. The absorbances of the resulting solutions were measured at 734 nm using a spectrophotometer (Benchmark Plus, Bio-Rad, Hercules, CA, USA).

To measure the DPPH radical scavenging activity, 100 μL aliquots of EEBC solutions at different concentrations were mixed with 100 μL DPPH solution (0.15 mM in methanol). The reaction mixture was incubated for 30 min in the dark at room temperature. The absorbances of the resulting solutions were measured at 517 nm. The radical scavenging capacities of the tested samples were calculated using the following equation:

Scavenging activity (%) = {1 − (Absorbance of sample/Absorbance of control)} × 100(4)

### 4.9. Aβ Aggregation Assay

Aβ (1–42) aggregation was measured using the SensoLyte^®^ Thioflavin T-amyloid aggregation kit (AnaSpec, Fremont, CA, USA), according to the manufacturer’s instructions. Briefly, thioflavin T was dissolved in assay buffer and used at a concentration of 100 µM. Samples were dissolved in assay buffer to make various concentrations. To determine the inhibition rate (%) of Aβ (1–42) aggregation in 96-well black microplates, the sample (5 µL) and Aβ (1–42) (85 µL) were mixed, followed by the addition of thioflavin T (10 µL). Fluorescence of thioflavin T was measured at intervals of 20 min for 2 h, with an excitation wavelength (λ_ex_) of 440 nm and an emission wavelength (λ_em_) of 485 nm using a SpectraMax i3 Multi-Mode Detection Platform (Molecular Devices, Sunnyvale, CA, USA). Fluorescence readings were expressed in relative fluorescence units. Assays were performed in triplicate and repeated three times. Morin (100 µM) was used as a positive control for inhibiting Aβ aggregation [59].

### 4.10. AChE and BChE Activity Assay

In vitro AChE activity was assessed according to a protocol based on Ellman’s colorimetric method [60], with modifications, using an Acetylcholinesterase Assay Kit (Abcam, Cambridge, UK). The stock solutions of EEBC and four marker compounds were dissolved in dimethyl sulfoxide at a concentration of 100 mg/mL or 100 mM, respectively. Assay samples were diluted with 0.1 M sodium phosphate buffer (pH 8.0). The AChE stock solution was prepared by dissolving 25 U/mL of 0.1% bovine serum albumin/H_2_O in 0.1 M sodium phosphate buffer (pH 7.3, assay buffer), to a final concentration of 35.2 mU/mL, before the assay. The substrates acetylthiocholine iodide and 5,5′-dithiobis-2-nitrobenzoic acid (DTNB) were dissolved in H_2_O and assay buffer, respectively, to make final concentration of 10 mM. For assays, 0.25 mL of 10 mM acetylthiocholine iodide and DTNB were mixed in 4.75 mL of assay buffer to a final concentration of 0.5 mM and used as the reaction mixture. For the enzymatic reaction in 96-well plates, 50 µL of the sample solution and 50 µL of the reaction mixture were mixed and preincubated for 10 min at room temperature. AChE solution (10 µL) was then added to initiate the reaction, which was performed for 1 h at room temperature. In vitro BChE activity was performed according to the manufacturer’s protocol using a Butyrylcholinesterase Activity Kit (BioVision, Milpitas, CA, USA) in a similar method to the AChE assay. The absorbance was measured at 412 nm using an Epoch microplate spectrophotometer (Bio-Tek Instruments, Winooski, VT, USA). The inhibition rate (%) of AChE activity was calculated by comparing the rate of reaction of the sample to that of the blank. All assays were performed in triplicate and repeated three times. Berberine was used as a positive control for AChE and BChE inhibition [61].

### 4.11. Statistical Analysis

All experiments were performed in triplicate. The data are expressed as mean ± standard error of the mean. Data were analyzed to determine differences between control and test groups using one-way analysis of variance and Dunnett’s multiple comparisons test. Statistical analysis was conducted with GraphPad Prism 7.0 (Graphpad Software, San Diego, CA, USA). *p* < 0.05 was considered statistically significant. EC_50_ or IC_50_ values were calculated using the SigmaPlot 10.0 (Systat Software, Chicago, IL, USA).

## Figures and Tables

**Table 1:** Linear range, regression equation, correlation coefficients, limits of detection (LODs), and limits of quantification (LOQs) for compounds.

Compound	Linear Range (μg/mL)	Regression Equation (*y* = a*x* + b) ^a)^		*r* ^2^	LOD ^b)^ (μg/mL)	LOQ ^c)^ (μg/mL)
		Slope (a)	Intercept (b)			
Gallic acid	6.25–200	25,130	1562.7	1.0000	0.726	2.199
(+)-catechin	12.5–400	8599.9	−4988.9	1.0000	0.381	1.154
Ellagic acid	3.125–100	104,400	−22,602	1.0000	0.315	0.954
Quercitrin	3.125–100	25,051	−4781.8	1.0000	0.172	0.520

^a)^ y = ax + b, y means peak area and x means concentration (μg/mL); ^b)^ LOD (Limit of detection): 3.3 × (SD of the response/slope of the calibration curve); ^c)^ LOQ (Limit of quantitation): 10 × (SD of the response/slope of the calibration curve).

**Table 2:** The contents of four compounds in *B. coccinea*.

Compound	Contents (mg/g)
Gallic acid	11.757 ± 0.012
(+)-catechin	18.736 ± 0.034
Ellagic acid	2.912 ± 0.001
Quercitrin	3.897 ± 0.004

## Data Availability

The data presented in this study are available within the article and Supplementary materials.

## Supplementary Materials

The following is available online at https://www.mdpi.com/article/10.3390/plants10040702/s1. Table S1: BChE assay results for EEBC and four marker compounds.
