# Supplementary material for: Simultaneous Quantification of Four Marker Compounds in Bauhinia coccinea Extract and Their Potential Inhibitory Effects on Alzheimer’s Disease Biomarkers
Source: Plants (Basel). 2021 Apr 6;10(4):702. doi: 10.3390/plants10040702 (PMC8067434; doi:10.3390/plants10040702)
Supplement: Supplementary file 1 [file plants-10-00702-s001.pdf]

Table S1. BChE assay results for EEBC and four marker compounds.

Samples	Conc. (µM)		OD			% Inhibition		Mean (%)	SD	SEM
Berberine	50	1.37	1.34	1.33	56.74	57.56	57.88	57.39	0.59	0.34
Gallic acid	6.25	3.20	3.21	3.13	-1.40	-1.53	0.94	-0.66	1.39	0.80
	12.5	3.18	3.20	3.13	-0.52	-1.34	0.94	-0.31	1.15	0.67
	25	3.09	3.09	3.08	2.33	2.27	2.65	2.42	0.20	0.12
	50	3.10	3.12	3.09	1.92	1.13	2.33	1.79	0.61	0.35
	100	3.12	3.08	3.08	1.19	2.58	2.65	2.14	0.82	0.48
Quercitrin	6.25	3.18	3.17	3.15	-0.64	-0.42	0.43	-0.21	0.57	0.33
	12.5	3.20	3.14	3.10	-1.37	0.75	1.86	0.41	1.64	0.95
	25	3.17	3.12	3.03	-0.17	1.26	4.07	1.72	2.16	1.25
	50	2.97	3.13	3.11	6.03	1.07	1.60	2.90	2.73	1.57
	100	3.10	3.05	3.12	1.83	3.47	1.16	2.15	1.19	0.69
(+)-Catechin	6.25	3.11	3.15	3.14	1.51	0.34	0.78	0.88	0.59	0.34
	12.5	3.09	3.13	3.14	2.11	1.10	0.78	1.33	0.69	0.40
	25	3.07	3.12	3.12	2.90	1.32	1.22	1.81	0.94	0.54
	50	3.09	3.11	3.12	2.36	1.48	1.16	1.67	0.62	0.36
	100	3.04	3.10	2.98	3.76	2.01	5.75	3.84	1.87	1.08
Ellagic acid	6.25	3.03	3.05	3.08	4.20	3.53	2.55	3.43	0.83	0.48
	12.5	3.05	3.02	3.04	3.53	4.36	3.72	3.87	0.43	0.25
	25	3.06	3.01	3.04	3.09	4.64	3.76	3.83	0.78	0.45
	50	3.08	3.05	3.07	2.46	3.41	2.93	2.93	0.47	0.27
	100	3.07	3.05	3.08	2.93	3.44	2.43	2.93	0.51	0.29
EEBC	6.25 (µg/mL)	3.45	3.45	3.48	0.88	0.91	-0.15	0.55	0.61	0.35
	12.5	3.45	3.42	3.47	0.85	1.54	0.22	0.87	0.66	0.38
	25	3.40	3.43	3.44	2.18	1.46	0.94	1.52	0.62	0.36
	50	3.41	3.44	3.48	1.89	1.14	0.02	1.02	0.94	0.54
	100	3.45	3.47	3.49	0.88	0.13	-0.30	0.24	0.60	0.34
